# How Do Hexokinases Inhibit Receptor-Mediated Apoptosis?

**DOI:** 10.3390/biology11030412

**Published:** 2022-03-08

**Authors:** Axel Schoeniger, Philipp Wolf, Frank Edlich

**Affiliations:** Institute of Biochemistry, Faculty of Veterinary Medicine, University of Leipzig, 04103 Leipzig, Germany; axel.schoeniger@vmf.uni-leipzig.de (A.S.); philipp.wolf@vmf.uni-leipzig.de (P.W.)

**Keywords:** cell death, BCL-2 proteins, cancer, immunotherapy, BH3 profiling

## Abstract

**Simple Summary:**

In multicellular animals, cells autonomously respond to lethal stress by inducing cell death programs. The most common regulated cell death is apoptosis. Cells protect their neighbors from damage by their cell contents or infection through this process. Apoptosis can occur as a result of intrinsic stress or induced by surface receptors, for example, by immune cells. In most cases, receptor-mediated apoptosis also requires the intrinsic signaling pathway. Intrinsic apoptosis is controlled by proteins of the B-cell lymphoma 2 (BCL-2) family. Pro-apoptotic BCL-2 proteins are inhibited by retrotranslocation from the mitochondria into the cytosol until the cell commits to apoptosis. Increasingly, discoveries show that BCL-2 proteins are regulated by proteins that are not themselves members of the BCL-2 family. Here, we discuss the selective inhibition of the link between death receptors activation and intrinsic apoptosis by hexokinases. These enzymes funnel glucose into the cellular metabolism. Independently, hexokinases retrotranslocate BCL-2 proteins and thereby protect cells from receptor-mediated apoptosis.

**Abstract:**

The regulated cell death apoptosis enables redundant or compromised cells in ontogeny and homeostasis to remove themselves receptor-dependent after extrinsic signaling or after internal stress by BCL-2 proteins on the outer mitochondrial membrane (OMM). Mitochondrial BCL-2 proteins are also often needed for receptor-mediated signaling in apoptosis. Then, the truncated BH3-only protein BID (tBID) blocks retrotranslocation of the pro-apoptotic BCL-2 proteins BAX and BAK from the mitochondria into the cytosol. BAX and BAK in turn permeabilize the OMM. Although the BCL-2 proteins are controlled by a complex regulatory network, a specific mechanism for the inhibition of tBID remained unknown. Curiously, it was suggested that hexokinases, which channel glucose into the metabolism, have an intriguing function in the regulation of apoptosis. Recent analysis of transient hexokinase interactions with BAX revealed its participation in the inhibition of BAX and also BAK by retrotranslocation from mitochondria to the cytosol. In contrast to general apoptosis inhibition by anti-apoptotic BCL-2 proteins, hexokinase I and hexokinase 2 specifically inhibit tBID and thus the mitochondrial apoptosis pathway in response to death receptor signaling. Mitochondrial hexokinase localization and BH3 binding of cytosolic hexokinase domains are prerequisites for protection against receptor-mediated cell death, whereas glucose metabolism is not. This mechanism protects cells from apoptosis induced by cytotoxic T cells.

## 1. Pro-Apoptotic BCL-2 Activities Control the Molecular Decision to Apoptosis

Superfluous, infected, or damaged cells remove themselves from the organism through apoptosis [1,2,3]. This cell death program is essential for the survival of multicellular animals and the most important protective mechanism against tumor development. Apoptotic cells fragment into vesicles termed “apoptotic bodies” which are completely eliminated by phagocytosis [4]. Thereby, the dying cell protects neighboring cells from their harmful contents by being removed from the body.

Intrinsic apoptosis is the most common form of programmed death, involving the permeabilization of the outer mitochondrial membrane (OMM) [5]. This pathway is governed by proteins of the B-cell lymphoma-2 (BCL-2) family (Table 1). The pro-apoptotic activities of BCL-2-associated X protein (BAX) and BCL-2 antagonist killer 1 (BAK) can permeabilize the OMM. Then, proteins from the mitochondrial intermembrane space (IMS) such as cytochrome *c* (cyt *c*) are released, which initiates the caspase (cysteinyl aspartate protease) cascade that disassembles the cell [6,7,8]. Therefore, BAX/BAK activation is the first irreversible step in intrinsic apoptosis. However, reduced or inhibited caspase activation can lead to cell survival despite OMM permeabilization [9,10]. Limited OMM permeabilization of a subset of mitochondria has also been demonstrated to be insufficient to induce apoptosis [11,12]. Thus, cells can bypass commitment to apoptosis after BAX/BAK activation. The efficient activation of the caspase cascade is the principal function of the BCL-2 proteins. If the underlying mechanisms are impaired, a pathway for tumor formation will be cleared. Incidentally, this is also true when tumor therapy fails to induce apoptosis in targeted cells.

In addition to cell intrinsic signaling, extracellular death receptor ligands can trigger apoptosis. Cytotoxic T cells eliminate tumor cells by this mechanism, which is started or enhanced by immunotherapy. Extracellular domains of transmembrane receptors such as Fas (CD95) bind to their trimeric ligands. The apoptotic signal is transmitted by the clustering of activated receptors and the formation of an intracellular adaptor protein scaffold. Within this scaffold, the initiator caspase-8 self-activates and initiates the caspase cascade by substrate cleavage. Processing of one of the caspase-8 substrates, the BCL-2 homology domain 3 (BH3)-only protein BID, links the death receptor apoptosis pathway to OMM permeabilization. Truncated BID (tBID) can inhibit pro-survival BCL-2 proteins, thereby activating the pro-apoptotic BCL-2 proteins BAX and BAK [13,14,15]. BAX/BAK-dependent permeabilization of the OMM is often necessary to trigger apoptosis by extrinsic signals [5]. Even cells competent of undergoing apoptosis by receptor-mediated caspase activation alone show a greatly enhanced apoptotic response by BAX or BAK [16,17].

## 2. Dynamic Retrotranslocation Determines the Effective BCL-2 Protein Pool

BAX and BAK are antagonized by the large group of anti-apoptotic BCL-2 proteins with high functional redundancy, including BCL-2, B-cell lymphoma-extra large (BCL-xL), and myeloid cell leukemia 1 (MCL-1). The BCL-2 protein fold, shared by both pro-apoptotic and anti-apoptotic family members, creates a hydrophobic surface groove that is occupied by the C-terminal transmembrane domain (TMD) in the cytosolic forms of BAX and BCL-xL [18,19]. Intermolecular interactions between BCL-2 proteins are primarily mediated by binding of a BH3 domain to another protein’s hydrophobic groove [20]. One precondition for interaction between BCL-2 proteins, however, is the presence of a lipid membrane [15]. In cells, BCL-2 proteins localize predominantly to the OMM depending on their TMDs and interact with this membrane [21].

**Figure 1:**
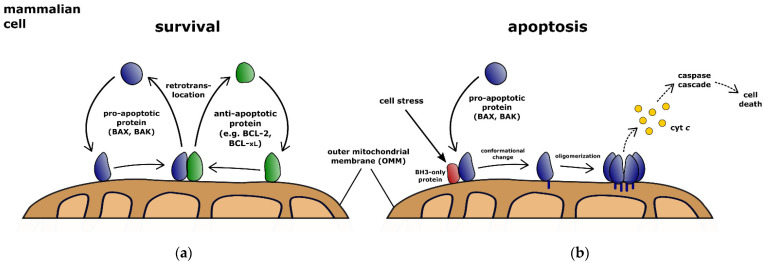
BCL-2 proteins inhibit apoptosis or commit the cell to mitochondrial apoptosis. (**a**) Pro-apoptotic BCL-2 proteins BAX and BAK (blue) constantly translocate to the outer mitochondrial membrane (OMM). After a change in the protein conformation of BAX or BAK the pro-apoptotic BCL-2 proteins are recognized by anti-apoptotic BCL-2 proteins, e.g., BCL-xL (green), and retrotranslocate back into the cytosol due to transient interactions between the two types of BCL-2 proteins. Retrotranslocation stabilizes the inactive forms of BAX and BAK and prevents, therefore, the activation of BAX or BAK in cells. (**b**) Lack of retrotranslocation of BAX or BAK commits the cell to apoptosis. BAX or BAK undergo further conformational changes at increased mitochondrial dwell times, oligomerize and permeabilize the OMM. The subsequent release of intermembrane space proteins, such as cytochrome *c* (cyt *c*), initiates the caspase cascade that dismantles the cell.

To this end, BAX and BAK translocate permanently to the OMM [22,23]. At the OMM, anti-apoptotic BCL-2 proteins inhibit BAX and BAK by constant retrotranslocation into the cytosol (Figure 1) [22,24,25,26]. Different shuttling rates of BAX and BAK lead to their distinct localization in the cell [25,27,28]. The shuttling of BAX and BAK establishes an equilibrium between protein pools in the cytosol and on the mitochondria. At the same time, only the mitochondrial population of proteins is available for activation [28]. Retrotranslocation, therefore, determines the cellular response to apoptosis stimulation [29,30,31]. BCL-2 proteins are also found in other compartments of the cell, yet only retrotranslocation between mitochondria and cytosol has been studied. For this purpose, the transition out of the membrane seems to be necessary [32]. Theoretically, the transition between other compartments and the mitochondria is also conceivable. To the ER, BCL-2 proteins could pass through the cytosol via retrotranslocation on the one hand and via lateral sorting from the mitochondria on the other hand [33,34]. In principle, it remains open whether BCL-2 proteins can be transported exclusively via the cytosol or also directly between different compartments. BAX/BAK retrotranslocation itself depends on recognition of exposed BH3 motifs by the hydrophobic groove of the pro-survival BCL-2 proteins [28]. BH3-only proteins, such as tBID, inhibit BCL-2 protein-dependent retrotranslocation of BAX and BAK [22]. Consequently, the presence of BH3-only proteins slows down the inhibitory retrotranslocation of BAX and BAK. The resulting increase in effective mitochondrial protein pools and prolongation of the dwell time of BAX and BAK molecules at the OMM increases the probability for apoptosis induction [28]. In other words, more stress means more BAX at the OMM and thus a greater chance for full activation of caspases.

## 3. Membrane Receptors Guide the Function of BCL-2 Proteins by Activation of Downstream GTPases

Cellular commitment to apoptosis is directed by the activity and localization of BCL-2 proteins. In addition to regulatory interactions between members of the BCL-2 family, proteins outside this family participate in apoptosis regulation. There is increasing evidence that the superfamily of GTPases interacts with various members of the BCL-2 family on different molecular levels. GTPases are characterized by their activation via GTP binding and their subsequent deactivation after GTP hydrolysis by an intrinsic enzyme activity [35]. Based on their structure, different subfamilies were identified within the GTPase superfamily such as the heterotrimeric G proteins (large GTPases) and small Ras-like GTPases [36].

G protein signaling is heavily tied to the activity of a prominent class of plasma membrane-embedded proteins, the G protein-coupled receptor (GPCR) family, which engage intracellular G proteins upon ligand binding [37,38]. Despite the huge number of different GPCRs, only a limited set of G proteins is available to guide (multi-) cellular survival [39,40]. Activation of G proteins by different GPCRs such as the angiotensin II receptor type 1, the vasopressin receptor 2, and the *N*-formyl peptide receptor induces the activation of executioner caspase-3, leading to apoptosis [41]. This pro-apoptotic effect was negatable by homologous receptor desensitization, shutting off G protein signaling [41]. Interestingly, cleavage of arrestin-2, an adaptor protein mediating homologous desensitization, by different caspases, reduced cellular resistance to apoptosis and enhanced tBID-mediated release of cyt *c* from mitochondria [42]. This indicates a feedback loop, balancing pro- and anti-apoptotic effects, as well as a temporal scale of G protein signaling, determining its pro- and anti-survival actions. Yet, this seems to strongly depend on the cellular system, which is reflected by controversial reports of pro- and anti-apoptotic G protein signaling as demonstrated for the muscarinic receptor M1 [43,44].

**Figure 2:**
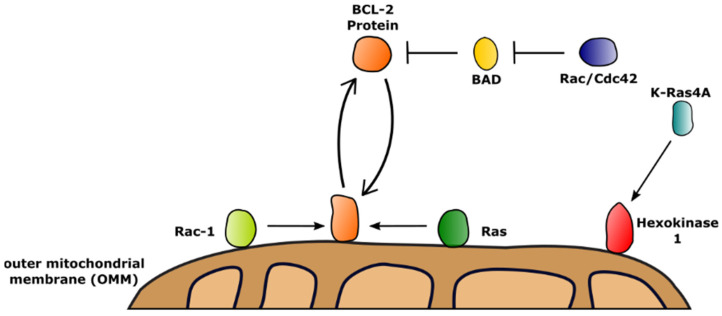
Interactions of small GTPases with the BCL-2 family, influencing BCL-2 protein function. Anti-apoptotic BCL-2 proteins such as BCL-2 (orange) shuttle between the cytoplasm and the outer mitochondrial membrane (OMM). BCL-2 protein function is regulated by members of the GTPase family. GTPases from the Rac and Ras subfamilies (light green and green, respectively) can bind to OMM-integrated BCL-2, enhancing its anti-apoptotic function. The anti-apoptotic effect of BCL-2 function is enhanced by inhibition of BAD through phosphorylation in a Rac/Cdc42 (dark blue)-dependent manner. Further, GTPases such as K-Ras4A (light blue) activate hexokinase I (red) on the OMM, participating in apoptosis regulation.

In contrast to the small family of heterotrimeric G proteins, which are characterized by their enzymatic active Gα subunits, the Ras-like GTPases contain 167 monomeric members, which are homologous to Gα [45,46]. A summary of known interactions between small GTPases and BCL-2 family members is given in Table 2 and Figure 2. One way of regulating cellular survival by small GTPases is by activation of downstream kinases as shown for the Ras effector kinase Raf-1. Activated Raf-1 subsequently regulates BCL-2 protein function by interaction with and phosphorylation of the BH3-only proteins BAD and BIM, interfering with their binding to anti-apoptotic BCL-2 and BCL-xL [47,48,49]. Similarly to the Ras subfamily, members of the Rho subfamily of GTPases can protect cells from committing to apoptosis by regulation of BAD/BCL-xL interactions via downstream kinase signaling as shown for Rac and Cdc42 [50].

In addition to employing downstream effectors to modulate cell survival, small GTPases also regulate BCL-2 proteins by direct interactions, potentially linking metabolic pathways to cellular survival [51]. Rac-1, a crucial participant in insulin-dependent glucose uptake and metabolism, was shown to interact with BCL-2 on mitochondria, increasing the pro-survival effects of the BCL-2 protein [52,53]. Besides Rac-1, the GTPase Ras engages the N-terminal BH4 domain of BCL-2 at the OMM using its C-terminal CAAX motif, which enables BCL-2 to suppress the apoptotic influence of Ras signaling [54].

**Table 2:** Overview of BCL-2 family interactions with the superfamily of small GTPases, regulating cellular commitment to apoptosis.

GTPase Superfamily	Mode of Action
Ras	Raf-1-dependent phosphorylation of pro-apoptotic BAD/BIM [47,48,49]
	Ras binding to BCL-2, increasing its anti-apoptotic effect [54]
	Activation of hexokinase I by K-Ras4A binding [55]
Rho	Rac-1 binding to BCL-2, increasing its anti-apoptotic effect [50]
	PAK-dependent phosphorylation of pro-apoptotic BAD by Rac/Cdc42 [50]

Generally, large GTPases such as the heterotrimeric G proteins and small GTPases such as Ras balance a vast field of signaling pathways, impacting cellular homeostasis. Their participation in various metabolic pathways indicate an important role in guiding cellular survival. For example, the K-Ras isoform K-Ras4A was recently found to directly interact with hexokinase I and hexokinase II, key enzymes of glucose metabolism, on the OMM. Whereas K-Ras4A was able to bind to hexokinase II, it failed to increase its enzymatic activity [55]. Contrary, binding of K-Ras4A to hexokinase I increased the enzymatic activity of hexokinase I [55].

## 4. Hexokinases: At the Crossroads between Glucose Metabolism and Apoptosis

Hexokinases phosphorylate hexose sugars, primarily glucose, trapping glucose within the cytoplasm and keeping the intracellular concentration of plain glucose low. Hexokinases, therefore, play a critical role in cellular uptake and disposition of glucose by committing glucose to glycolytic and pentose phosphate pathways or storage. In mammalian tissues, four isoforms of hexokinases (I–IV) are found. They are constitutively expressed in most tissues but differ significantly in their tissue-specific distribution, subcellular localization, and functional properties (Table 3).

There is growing evidence that there is a direct link between glucose metabolism and apoptosis. First discoveries showed physical interactions between hexokinases and mitochondria [67]. It was later found that mainly hexokinase isoforms I and II bind to the mitochondria [68]. The subcellular distribution of hexokinase II has been reported to be dependent on glucose availability, whereas the distribution of hexokinase I is unaffected by varying glucose levels [69]. The interaction of hexokinases I and II with the mitochondria is facilitated by an N-terminal binding motif containing a short hydrophobic α-helix that is likely to be inserted into the OMM [60,70,71]. Truncated hexokinase lacking this hydrophobic region is unable to bind to the mitochondria [72]. In fact, it has been shown that a single mutation at the N-terminal domain of hexokinase II is enough to prevent binding to the OMM [73]. Hexokinases III and IV lack this hydrophobic N-terminal domain, and thus do not interact with the mitochondria.

Due to their binding to the OMM, hexokinases I and II have been linked to apoptosis. Hexokinase II has been shown to inhibit indomethacin-induced cyt *c* release and caspase-3 activation by preventing BAX from binding to the mitochondria [74]. Overexpression of hexokinase I has been shown to inhibit staurosporine-induced apoptosis [75]. Furthermore, overexpression of hexokinase I or II seems to decrease stress-induced accumulation of mitochondrial BAX, leading to the suggestion that hexokinases and BAX may compete for common binding sites on the mitochondria [76]. However, activation of BAX, as indicated by exposure of the carboxy-terminal 6A7 epitope, was not affected [76]. It has thus been hypothesized that hexokinase II sequesters active BAX in the cytosol, although no such interaction could be detected by immunoprecipitation [76]. The anti-apoptotic effect of hexokinase II is in line with a hexokinase II upregulation observed in many types of cancer [77]. Consequently, it has been suggested that overexpression of hexokinases in tumor cells contributes to resistance against chemotherapeutic drugs [78,79,80]. However, a survival advantage of tumor cells could not only be attributed to the inhibition of apoptosis by hexokinases but also to an increased rate of glycolysis and perhaps ATP production. Further studies showed that the anti-apoptotic effect of hexokinases is significantly reduced when truncated hexokinase isoforms that lack the N-terminal mitochondrial binding domain are overexpressed [72]. Interestingly, similar results have been obtained with full-length but catalytically inactive forms of both hexokinase I and II. These effects could either result from impaired hexokinase localization or the lack of glucose conversion to glucose-6-phosphate. In contrast, knockdown of hexokinase I has been reported to increase mitochondrial BAX and to promote TNF-induced BAX oligomerization [81]. In addition, hexokinase II knockdown has been suggested to enhance the expression of BAX and caspase-3, while BCL-2 could be downregulated [82]. Further studies revealed that depletion of hexokinase II decreases cancer cell proliferation and increases sensitivity to cell death inducers [58,83]. Effects similar to the hexokinase II knockdown have been observed when this enzyme gets displaced from mitochondria or from interaction sites between mitochondria and ER (mitochondria-associated membranes; MAMs) by selective peptides [59,84]. Pro-apoptotic effects could also be achieved by detaching mitochondrial-bound hexokinase I with clotrimazole [74,81].

Specific binding of hexokinases to the mitochondria is mediated by the voltage-dependent anion channel (VDAC), which is the major transport channel mediating the passage of ions and metabolites across the OMM [85,86,87,88]. The interaction with VDACs is thought to provide hexokinases preferred access to mitochondrially generated ATP [62]. Three isoforms of VDAC have been identified, VDAC1, VDAC2, and VDAC3 [89]. Different degrees of colocalization between these isoforms and hexokinase I have been revealed by STED microscopy [90]. Moreover, evidence suggests that distinct hexokinase I pools exist on the mitochondria that are not colocalized with any of the isoforms [90]. Although hexokinases seem to bind to the mitochondria without VDAC, knockout of VDAC results in a significant decrease in mitochondrial hexokinase [91].

The structural basis of complex formation between VDAC and hexokinases is yet to be elucidated. It has been suggested that hexokinase first inserts into the OMM and then interacts with VDAC on the outer leaflet of OMM [92]. Complex formation between both proteins seems to be mediated by their N-terminal domains [93,94]. Removal of the N-terminal domain of hexokinases or VDACs abolishes their interaction [93,95]. Studies show that their association protects cells against apoptosis [74,75]. A single mutation is sufficient to largely abolish hexokinase I binding to VDACs and prevent hexokinase I-mediated protection from cell death [57]. Interaction of hexokinases and VDACs has also been implicated in aerobic glycolysis (“Warburg effect”) and proliferation of tumor cells [96]. Strikingly, hexokinases have been reported to bind to VDACs more tightly in cancer cells compared to control cells [96]. However, the molecular basis of hexokinase-mediated apoptosis inhibition remained unresolved. VDAC is believed to adopt a closed state upon activation of apoptosis [97]. It has been suggested that binding of hexokinase to VDACs leads to channel opening [98,99]. There is also evidence, however, indicating that hexokinase I induces VDAC1 closure (“low-conducting” state), leading to diminished metabolic exchange [95,100,101]. Thus, hexokinase-dependent VDAC closure has been speculated to be an anti-apoptotic event by preventing cyt *c* release [95]. These discrepancies may be explained by the assumption that VDACs, besides an open and closed state, can adopt a partially closed conformation through their interaction with hexokinases [92]. This conformation would still allow some flux of low molecular weight substances while preventing the release of apoptotic factors. Interestingly, it has been shown that hexokinase-mediated VDAC closure can be reversed by glucose-6-phosphate [95,102], indicating a direct link between glucose metabolism and apoptosis perhaps through the influence of hexokinase localization on apoptosis. It has been further suggested that closing of VDACs by hexokinases could be inhibited by AKT signaling and overexpressed BCL-2 or BCL-xL [75,97]. However, opposing effects were also reported [64]. Nonetheless, a competition between hexokinases and BCL-2 proteins for VDAC binding sites has been proposed [63]. This assumption is consistent with the recent observation that under high glucose condition the interaction between VDAC1 and BAX is enhanced [103]. These effects could be partly reversed by overexpression of hexokinase II [103].

In addition, other binding partners of hexokinase II have been identified. The Tp53-induced Glycolysis and Apoptosis Regulator (TIGAR) is a p53 target gene. TIGAR has been shown to translocate to the mitochondria under hypoxic conditions, where it forms a complex with hexokinase II and increases hexokinase activity [104]. Similarly, the phosphoprotein enriched in astrocytes (PEA15) binds to hexokinase II following hypoxia and seems to increase anti-apoptotic effects [105]. These findings suggest that hexokinase II interacts with both ubiquitously and tissue-specific expressed binding partners.

Another regulator of hexokinases is glycogen synthase kinase 3β (GSK3β), which is a downstream effector of the phosphatidylinositol 3-kinase (PI3K)/AKT signaling pathway. GSK3β has been suggested to promote apoptosis [56,61]. Activation of GSK3β seems to induce the dissociation of hexokinase II from the OMM via phosphorylation of VDAC [74,106]. Inhibition of GSK3β has been shown to increase hexokinase II binding to the mitochondria and to protect against rotenone-induced apoptosis [106]. In contrast to the GSK3β-mediated disruption of hexokinase/VDAC interaction, phosphorylation of VDAC by PKC-ε has been reported to promote hexokinase binding [72,107]. These findings indicate an intricate regulatory web for the complex formation of hexokinases and VDACs.

## 5. Hexokinase-Dependent Retrotranslocation Protects Cells against Extrinsic Apoptosis

Although a role for hexokinases has been suspected in the regulation of mitochondrial apoptosis for some time, only recently the molecular link has been discovered [108]. Studying transient protein interactions with BAX, hexokinases I and II were identified. Owing to the pronounced regulation observed in tumors, hexokinase II is likely involved in cell death regulation. However, it shares mitochondrial association and probably the mechanism of binding to mitochondria and dissociation with hexokinase I. Hence, it is not too surprising that both hexokinases are involved in apoptosis control.

The analysis of transient hexokinase interactions with BAX revealed involvement in inhibition of BAX and also BAK by retrotranslocation from mitochondria to the cytosol. Hexokinases also accelerate BCL-xL retrotranslocation comparable to the anti-apoptotic BCL-2 protein MCL-1. In turn, overexpression of BCL-xL shifts the localization of hexokinases to the cytosol. In other words, there is evidence for a strong interdependence of the proteins, which retrotranslocate BAX and BAK. A function of hexokinases in retrotranslocation seems to imply that hexokinases can protect cells from apoptosis in general. However, this is not the case. In fact, inhibition of only receptor-mediated apoptosis by hexokinases can be observed. In a reduced cell system lacking the prominent members of the BCL-2 family, hexokinases alone inhibited BAX only to a modest extent [108]. A substantial effect of hexokinase was observed only when cell survival was dependent on the inhibition of tBID. The discrepancy between minimal direct effect of hexokinases on BAX activity and paramount role in specific inhibition of tBID-dependent receptor-mediated apoptosis led to the discovery of hexokinase-dependent retrotranslocation of tBID [108].

This function of the hexokinases is independent of the phosphorylation of glucose to glucose-6-phosphate. However, mitochondrial association of the hexokinases is essential for retrotranslocation. On the mitochondria hexokinases form complexes with VDAC2 similar to pro-survival BCL-2 proteins [32,109]. However, VDAC2 seems to either interact with pro-survival BCL-2 proteins or hexokinases. Neither BAX nor BAK are present in stable complexes of VDAC2 with pro-survival BCL-2 proteins or those VDAC2 complexes containing hexokinases. Thus, stable complexes between BCL-2 proteins in association with the OMM seem lacking. Only the interaction between tBID and BCL-xL seems to be an exception to this rule: BCL-xL decreases the rate of tBID retrotranslocation [108]. Therefore, the mitochondrial pool of both proteins increases due to OMM-embedded tBID/BCL-xL complexes [110,111]. By stabilizing common complexes, tBID appears to have the exceptional ability to reduce the effective BCL-xL protein pool for BAX/BAK retrotranslocation. It follows that death receptor signaling could be particularly effective in cells addicted to BCL-xL activity. The tBID-specific hexokinase-dependent retrotranslocation protects cells particularly from this apoptosis signaling axis.

**Figure 3:**
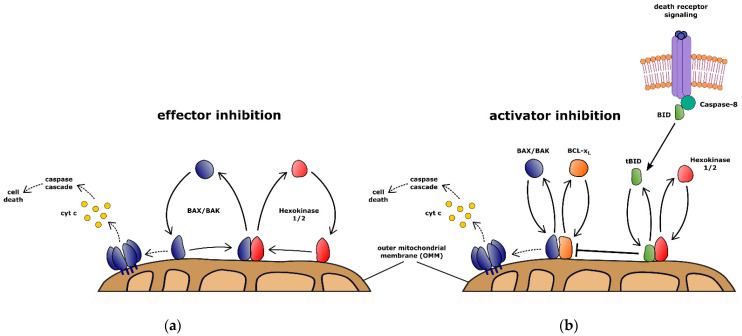
BCL-2 proteins inhibit apoptosis or commit the cell to mitochondrial apoptosis as a result of receptor-mediated apoptosis or intrinsic stress. (**a**) Hexokinase-dependent effector inhibition functions in analogy to BCL-2 protein-mediated retrotranslocation are involved in cell protection from any signal that can potentially trigger apoptosis. This mechanism is universally expected in mammalian cells but seems to be of lesser importance compared to the role of anti-apoptotic BCL-2 proteins. Constant OMM association of pro-apoptotic BCL-2 proteins (blue) is counteracted when BAX and BAK are recognized after a major conformational change by hexokinases (red) and retrotranslocated into the cytosol. In the absence of retrotranslocation of BAX or BAK, apoptosis is initiated through oligomerization of BAX and/or BAK and permeabilizes the OMM leading to cytochrome *c* (cyt *c*) release and caspase activity. Therefore, hexokinases prevent OMM permeabilization and commitment to apoptosis. (**b**) Additional inhibition of the activator tBID by hexokinases specifically protects cells from receptor-mediated apoptosis by cytotoxic T cells. In response to ligands death receptors, e.g., Fas or TRAIL-R, trimerize and initiate the formation of a caspase-8 (dark green) activating scaffold. Caspase-8 activity results in BID (green) cleavage. Mitochondrial tBID inhibits BAX/BAK (blue) retrotranslocation by competing with BAX and BAK for pro-survival BCL-2 protein, e.g., BCL-xL (orange), interactions. Therefore, tBID shifts BAX and BAK towards the active forms by forming OMM-embedded complexes with BCL-xL. Mitochondrial hexokinase I/II (red) selectively retrotranslocate tBID into the cytosol. Therefore, hexokinases prevent BAX/BAK activation in response to death receptor-mediated apoptosis.

The dependence on hexokinase localization and the specificity toward receptor-mediated apoptosis can be explanations as to why this mechanism remained hidden. Mitochondrial hexokinases inhibit apoptosis by effector inhibition through BAX/BAK retrotranslocation and activator inhibition by retrotranslocating mitochondrial tBID (Figure 3). Hexokinase-mediated BAX/BAK retrotranslocation potentially counteracts any mitochondrial apoptosis signaling. This process occurs in all mammalian cells and depends on the localization and expression of hexokinases I and II. Elevated levels of specifically hexokinase II in some tumor entities suggest a more important role. Increased glucose metabolism was observed to stabilize HKII glucose dependently. The resulting anti-apoptotic effect could thus contribute to the development of vascular complications of diabetes, diabetic embryopathy, and insulin resistance. This is also supported by increased HKII expression levels in cancer-associated adipose tissue [112]. Indeed, apoptosis is linked to decreased HKII levels and mitochondrial binding in obesity and type II diabetes [113].

Nevertheless, in experiments, hexokinases protected tumor cells significantly less than anti-apoptotic BCL-2 proteins [108]. Crucial to cell survival after death receptor signaling, however, is the retrotranslocation of tBID. Hexokinase-dependent retrotranslocation of tBID can prevent cell death in cells that require mitochondria for receptor-mediated apoptosis (type II). Even if mitochondrial signaling is not essential for receptor-mediated apoptosis (type I), the proportion of apoptotic cells is likely reduced. Thus, inhibition of the activator tBID reduces apoptosis triggered by cytotoxic T cells. This mechanism is also likely to be more pronounced in tumors with high hexokinase levels. Future studies should investigate the role of hexokinase-dependent retrotranslocation in immune evasion.

In addition to association with mitochondria, the cytosolic domains of hexokinases are also required for interaction and retrotranslocation of BCL-2 proteins. tBID retrotranslocation can be inhibited by BH3 mimetics. The role of the BH3 motif is supported by reduced interactions between hexokinases and BAX variants of the BH3 motif [108]. The retrotranslocation of BAX, BAK, and tBID, the inhibitory effect of BH3 mimetics, the binding to tBID, BIM, and BAX, and the disruptive effect of BAX-BH3 variants on hexokinases suggest that hexokinases interact with BCL-2 proteins via the BH3 motif. Binding of hexokinases to the BH3 motif may explain why hexokinases can retrotranslocate BAX, BAK, and tBID. Differences in the BH3 motif or secondary binding sites could impede BIM shuttling. Despite structural and functional differences, hexokinases and pro-survival BCL-2 proteins retrotranslocate BCL-2 proteins.

Hexokinase-dependent retrotranslocation of tBID is a direct countermeasure to death receptor-dependent initiation of OMM permeabilization. This additional layer of apoptosis regulation contributes to cell-to-cell differences in apoptosis induction [114]. Recently, it was suggested that tBID permeabilizes the OMM in the absence of BAX and BAK [115]. Indeed, such a special role of tBID among BH3-only proteins would explain why a tBID-specific inhibitory mechanism is necessary to protect the healthy cell. Hexokinase-dependent resistance to death receptor ligands, such as TRAIL and FasL, provides further complexity to the design of successful anti-tumor strategies. Apoptosis induction by cytotoxic T cells requires inhibition of hexokinase-dependent BCL-2 protein retrotranslocation when progression relies on caspase-mediated BID cleavage. Observations of cell type-specific differential apoptosis induction by tBID and BIM could be caused by differential hexokinase-dependent tBID retrotranslocation [116,117]. Varying hexokinase activities could create apparent differences in tBID and BIM activities in some cell types while lacking from others [118].

Hexokinases also directly inhibit commitment to apoptosis by BAX/BAK retrotranslocation. Strikingly, hexokinases enhance BAX/BAK retrotranslocation to the same rates as pro-survival BCL-2 proteins, while protection from BAX in cells lacking BCL-2 proteins is considerably lower [108]. Therefore, the dominant effect of apoptosis inhibition by hexokinases results from tBID shuttling. Hexokinase-dependent tBID retrotranslocation frees pro-survival BCL-2 proteins to retrotranslocate BAX and BAK into the cytosol in a ‘primed to death’ scenario [119,120]. BAX/BAK retrotranslocation especially following intrinsic stress-induced signaling is dominated by pro-survival BCL-2 proteins. Interestingly, BID has also been reported as a substrate of caspase-2 in response to ER stress or DNA damage signaling [65,66]. Nonetheless, hexokinase-dependent apoptosis inhibition seems specific to death receptor signaling [108].

## 6. Conclusions

The BCL-2 protein family interacts with a variety of different protein species to regulate the cellular fate. Hexokinases provide a link from glucose metabolism to cell survival. Mitochondrial hexokinases inhibit apoptosis by two different mechanisms: effector inhibition through BAX/BAK retrotranslocation and activator inhibition by tBID retrotranslocation specifically preventing mitochondrial apoptosis engagement following death receptor signaling. Hexokinase-dependent retrotranslocation safeguards, therefore, cells from apoptosis induced by cytotoxic T cells and could reduce apoptosis in obesity and type II diabetes.

## Figures and Tables

**Table 1:** Classification, role, and interactions of BCL-2 family members.

BCL-2 Family Member	Gene Name	Activity	Associated Diseases	Interacting BCL-2 Family Proteins in Cancer
BCL-2	BCL2	Anti-apoptotic	Follicular lymphoma 1,	BAX, BAD, BIM, tBID, PUMA
			high-grade B-cell lymphoma	
BCL-xL	BCL2L1	Anti-apoptotic	Absolute glaucoma,	BAX, BAK, BAD, BIM, tBID, PUMA
			tongue carcinoma	
MCL-1	MCL1	Anti-apoptotic	Myeloid leukemia,	BAX, BAK, BIM, tBID, NOXA, PUMA
			chlamydia	
BAX	BAX	Pro-apoptotic	T-cell acute lymphoblastic leukemia,	MCL-1, BFL-1, BCL-xL, BCL-2, BCL-w,
			colorectal cancer	BCL-B, PUMA, BIM, tBID
BAK	BAK1	Pro-apoptotic	Absolute glaucoma,	MCL-1, BFL-1, BCL-xL, PUMA, BIM, tBID
			keratoacanthoma	
BID	BID	Pro-apoptotic	Bladder transitional cell papilloma,	MCL-1, BFL-1, BCL-xL, BCL-2, BCL-w,
			colon adenocarcinoma	BCL-B, BAX, BAK
BIM	BCL2L11	Pro-apoptotic	Interleukin-7 receptor alpha deficiency,	MCL-1, BFL-1, BCL-xL, BCL-2, BCL-w,
			lymphoproliferative syndrome	BCL-B, BAX, BAK
BAD	BAD	Pro-apoptotic	B-cell lymphoma,	BCL-2, BCL-xL, BCL-w
			transient cerebral ischemia	

**Table 3:** Classification, functions, and interactions of hexokinases.

HK	Tissue Distribution	Subcellular Localization	Functions	Suggested Interactions in Cell Death Signaling	References
I	All mammalian tissues,	OMM, cytosol	Glucose catabolism,	BCL-xL	[56]
	main isoform in the brain		apoptosis regulator	BID	[56,57] *
				BIM	[56]
				BAX	[56]
				BAK	[56]
				VDAC	[58,59]
II	Heart, skeletal muscle,	OMM, cytosol	Glucose catabolism,	BAX	[56,60] *
	adipose tissue		glycogen synthesis,	BAK	[56]
			apoptosis regulator	VDAC	[58,59]
				PKCε	[61]
				AKT	[62]
				PEA15	[63]
				TIGAR	[64]
III	Ubiquitously expressed at low levels,	Perinuclear	Glucose catabolism		
	highest expression in lung, kidney	compartment			
	and liver				
IV	Liver, pancreatic islets, certain	Cytosol	Glucose catabolism,	BAD	[65]
	parts of the brain and gut		intracellular glucose sensor	VDAC	[66]

* Competitive interaction reported.; HK: hexokinase.
